# Effect of calcium dobesilate on intraretinal cysts and exudates in non-proliferative diabetic retinopathy: a Fiji ImageJ analysis

**DOI:** 10.1186/s12886-026-04750-8

**Published:** 2026-03-21

**Authors:** Bünyamin Kutluksaman, Ali Dal, Mehmet Çıtırık

**Affiliations:** 1 Department of Ophthalmology, Tayfur Ata Sökmen Faculty of Medicine, Hatay Mustafa Kemal University, Antakya, Hatay, 31060 Turkey; 2 Department of Ophthalmology, University of Health Sciences, Ankara Etlik City Hospital, Ankara, Turkey

**Keywords:** Diabetic retinopathy, Calcium dobesilate, Exudates, Intraretinal cysts, ImageJ, Fiji software

## Abstract

**Purpose:**

Diabetic retinopathy (DR) is a leading cause of vision loss in diabetic patients, primarily due to increased vascular permeability and microvascular dysfunction. Calcium dobesilate (CaD) has been suggested as a potential treatment to mitigate retinal exudates and intraretinal cyst formation. This study aims to quantitatively evaluate the effects of CaD on intraretinal cyst and exudate sizes in non-proliferative diabetic retinopathy (NPDR) using Fiji ImageJ software.

**Methods:**

This retrospective observational study included 132 NPDR patients aged 40–69, divided into two groups: those receiving CaD (*n* = 66) and those not receiving treatment (*n* = 66). Optical coherence tomography (OCT) was used to measure the sizes of intraretinal cysts (IRC) and intraretinal exudates (IRE) at baseline, 3, and 6 months. Image analysis was performed using Fiji ImageJ. Statistical comparisons were made using repeated measures ANOVA and non-parametric tests.

**Results:**

At 6 months, the treatment group exhibited a non-significant reduction in IRC and IRE sizes, while the control group showed a significant increase in both parameters (*p* = 0.002). No significant differences in best-corrected visual acuity (BCVA) were observed between the groups (*p* > 0.05).

**Conclusion:**

Although the reduction in IRC and IRE sizes in the CaD group was not statistically significant, the significant progression of these lesions in the control group suggests a potential protective effect of CaD. Further long-term prospective studies are warranted to validate these findings and determine the clinical significance of CaD in NPDR management.

## Introduction

Diabetes mellitus (DM) is a chronic condition characterized by microvascular and macrovascular complications, including diabetic retinopathy (DR), which is increasingly prevalent globally each year [1]. Long-term hyperglycemia causes oxidative stress, inflammation, and endothelial dysfunction, all of which are involved in the pathophysiological mechanism by which DR develops. DR develops in roughly 30–35% of patients diagnosed with DM globally, whereas complications associated with the potential for vision loss, such as diabetic macular edema (DME), occur in about 10% of cases [2]. 

DR may manifest in significant microvascular complications through a broad clinical range, from nonproliferative diabetic retinopathy (NPDR) to proliferative diabetic retinopathy (PDR) [3]. The primary pathophysiology involves the disruption of the blood-retinal barrier (BRB), a critical structure that maintains retinal integrity. Oxidative stress caused by long-term hyperglycemia damages endothelial cells and pericytes, causing permeability in the capillary inner BRB, and as a result, the inflammatory cascade is triggered [4, 5]. Damaged endothelial cells and their supporting pericytes cause microaneurysms, and exudates and vascular leaks occur due to the impaired capillary wall. As a result, increased mediators such as Vascular Endothelial Growth Factor (VEGF), Interleukin-6 (IL-6), Intercellular Adhesion Molecule-1 (ICAM-1), and Monocyte Chemoattractant Protein-1 (MCP-1) trigger the inflammatory process in the environment, promoting neovascularization and cell apoptosis [6]. 

In the NPDR stage, controlling systemic additional factors such as hyperglycemia, hypertension, and dyslipidemia, as well as optimizing retinal perfusion, is of utmost importance in order to retard the progression of the disease [7]. While strict screening and early interventions in the early stages may prevent or at least delay vision loss, invasive treatments such as anti-VEGF and corticosteroid intravitreal injections, laser photocoagulation, or surgery are usually needed in the advanced stages [3]. Although beneficial for advanced-stage targets, these procedures are generally inadequate to reverse significant damage; thus, early-stage management is crucial [8]. 

Therapeutic approaches focusing on oxidative stress, inflammation, and VEGF, since their main roles in diabetic retinopathy, continue to be relevant [9]. Apart from NPDR’s systematic management, drug and minimally invasive techniques targeting early-stage pathways are also significant [10]. Therefore, cost-effective and easily accessible treatment options are of great importance, especially in resource-limited health regions.

Calcium Dobesilate (CaD), which reduces vascular permeability and improves microcirculation by reducing oxidative stress and inflammation, is one such agents used in early-stage DR, and it is suggested that its effects in reducing blood viscosity and capillary permeability contribute to the formation of intraretinal cysts (IRCs) and exudates (IREs) [11, 12]. The clinical efficacy of this agent remains contentious, with literature presenting conflicting results [13–15]. 

While previous studies on CaD mostly focused on parameters such as macular thickness and visual acuity, in this study we aimed to examine early-stage structural changes such as IRC and IRE sizes in the NPDR stages. Image analyses for size assessment were performed using the Fiji ImageJ application developed as open-source software by the US National Institutes of Health (NIH) [16]. Fiji ImageJ, which can provide reliable area measurement capabilities and contains multiple advanced image processing algorithms, is an ideal tool for such analyses with its area measurement and image segmentation features [17]. We anticipate that our findings may be of clinical value in providing cost-effective and noninvasive treatment options for DR, particularly in underserved health areas.

## Materials and methods

**Fig. 1:**
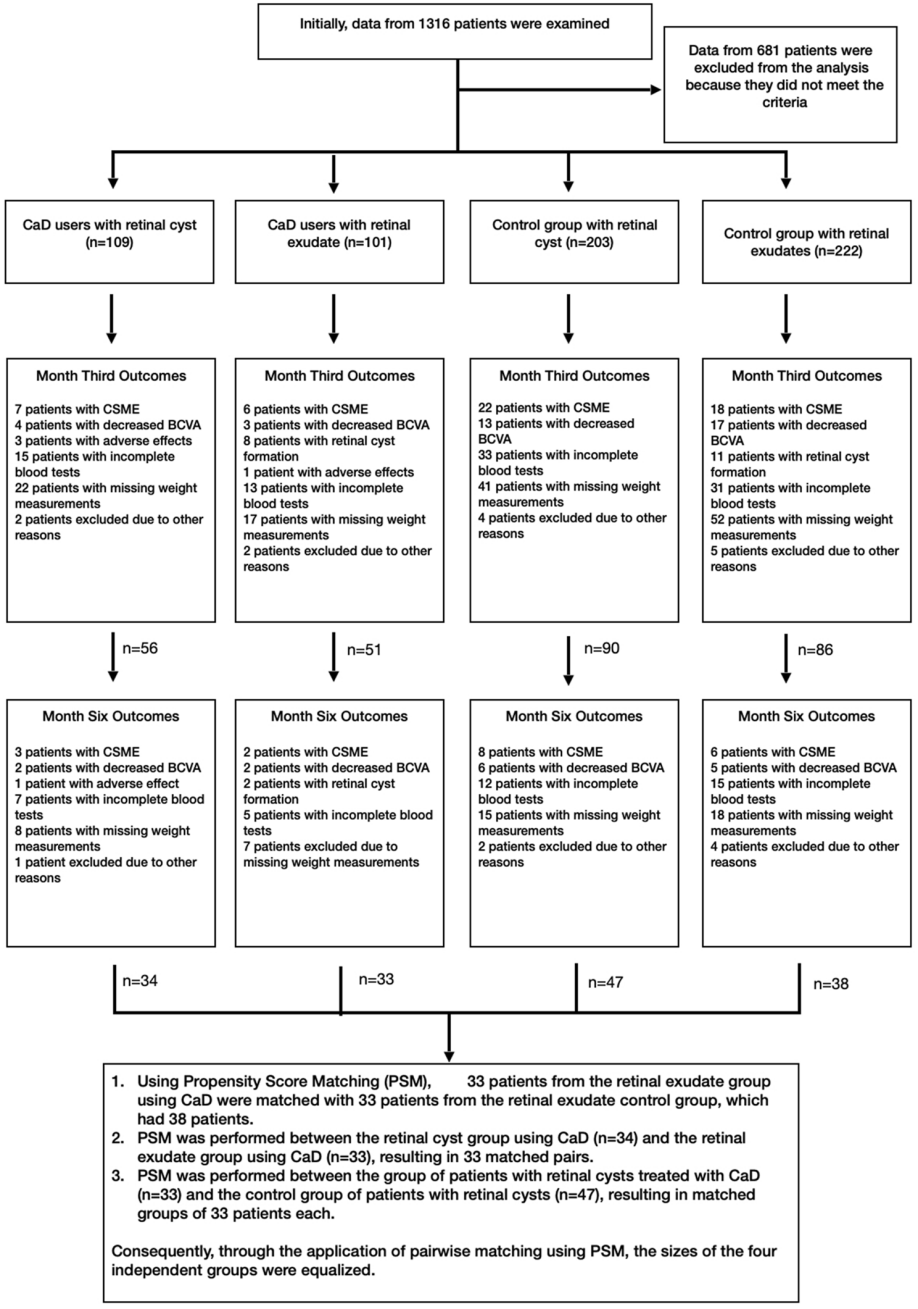
Algorithm for searching patient data to identify events such as death, surgical operation, laser photocoagulation, or mean arterial pressure exceeding the limit

This retrospective observational study was conducted in the Ophthalmology Clinic of Hatay Mustafa Kemal University. The records of patients aged 40–69 years who were diagnosed with adult-onset type 2 diabetes and were followed up in the retina clinic with mild-moderate non-proliferative diabetic retinopathy (NPDR) in at least one eye were examined. Approval was received from the University Ethics Committee for the study (decision number 18.12.2024-13-60), and the principles of the Declaration of Helsinki (revised in 2020) were followed. The specified inclusion and exclusion criteria were applied at each stage, and a total of 132 eyes of 132 patients were evaluated in the study (Fig. 1). The data of patients who did not use the medication after detailed information was evaluated as the control group. Initially, 1316 patient files were identified based on diagnosis and age range. These records were evaluated according to the inclusion and exclusion criteria at all follow-up time points. Following this assessment, the data of 132 eligible patients were subjected to propensity score matching, resulting in four matched groups, each comprising 33 patients.

Within the scope of the study, patients were divided into two main groups: those who received CaD treatment (*n* = 66) and those who did not (*n* = 66). Although this preference-based allocation introduced a potential selection bias, we retrospectively applied stringent inclusion and exclusion criteria to minimize its impact. Group homogeneity was sought by including only patients whose key systemic parameters—including HbA1c levels, blood pressure, and lipid profiles—were documented to be stable across all follow-up visits. Patients exhibiting systemic fluctuations during the study period were excluded from the final analysis to reduce confounding related to general metabolic instability. To preserve the validity of longitudinal structural analyses, specific clinical endpoints were predefined for censoring patients during follow-up. Progression to clinically significant macular edema (CSME) or center-involved diabetic macular edema (CI-DME), requiring rescue intravitreal therapy, was defined as a secondary clinical endpoint.

Patients meeting these criteria were treated with intravitreal anti-VEGF agents (e.g., ranibizumab or aflibercept) or corticosteroid therapy (dexamethasone implant), according to standard clinical practice. As such interventions are known to substantially alter IRC and IRE morphology, these patients were censored from further structural analyses at the time of rescue treatment to minimize confounding effects.

Each of these main groups was divided into subgroups according to the presence of IRC or IRE, creating a total of four groups: CaD users with IRC or IRE and non-users (*n* = 33 in each group). In patients with both IRC and IRE, only IRC was considered for evaluation. Clinical and imaging data of the patients before treatment and at 3 and 6 months after treatment were analyzed between these groups. Inclusion criteria for the study were first diagnosis of NPDR and type 2 diabetes, not having received oral CaD treatment before, accepting to use CaD treatment as oral tablets for 6 months (at a dose of 500 mg twice daily or 1000 mg once daily), detection of IRE and/or IRC by optical coherence tomography (OCT), absence of clinically significant diabetic macular edema (CSDME) or centrally involved diabetic macular edema, best corrected visual acuity (BCVA) of 0.6 (Snellen) or higher, HbA1c levels below 7.5%, regular blood tests during treatment, and no history of tobacco or alcohol consumption.

The exclusion criteria from the study were as follows: pregnancy or lactation, non-NPDR ophthalmic disease (e.g. glaucoma, cataract, etc.) or diagnosis of PDR, previous vitreoretinal surgery, laser photocoagulation, or intravitreal injection, corneal scarring, dense cataract, or conditions that prevent posterior segment imaging such as vitreous opacity, presence of vitreomacular traction or vitreous hemorrhage, irregular use of CaD during the six-month treatment period or discontinuation of treatment, HbA1c level above 7.5% during follow-up, liver or kidney dysfunction, any inflammatory/autoimmune ocular disease, dyslipidemia (total cholesterol > 200 mg/dL, LDL cholesterol > 130 mg/dL, HDL cholesterol < 40 mg/dL in men or < 50 mg/dL in women, triglycerides > 150 mg/dL, or despite lipid-lowering therapy failure to control these levels), uncontrolled hypertension (systolic blood pressure ≥ 140 mmHg or diastolic blood pressure ≥ 90 mmHg or failure to achieve these target values ​​despite antihypertensive therapy), a decrease in BCVA below 0.6 (Snellen) during follow-up, or need for or administration of intravitreal injections due to CSDME or centrally involved DME. Concomitant systemic treatments were documented and categorized for analysis as follows: antidiabetic regimens (insulin or oral antidiabetic drugs), antihypertensive medications (specifically RAAS inhibitors [ACE inhibitors and ARBs] vs. other classes), and lipid-lowering therapies (statins/fibrates vs. none). By applying these stringent exclusion criteria for uncontrolled systemic parameters and ensuring the stability of these categorized treatments, we aimed to isolate the specific pharmacologic effects of CaD and minimize potential confounding variables related to systemic metabolic fluctuations.

Under the heading of measurement of clinical and metabolic parameters, demographic data of the patients, such as age and gender, were recorded. Body weight was measured with a digital scale with a sensitivity of ± 0.1 kg, and height was measured with a stadiometer with a sensitivity of ± 0.1 cm, and body mass index (BMI) was calculated with the formula [weight (kg) / height² (m²)]. In laboratory analyses performed every three months, HbA1c, blood count, hemoglobin concentration, liver and kidney function tests, electrolyte levels, fibrinogen, and lipid fractions (total cholesterol, HDL, LDL, and triglycerides) were evaluated. Patients who fell outside the inclusion criteria determined for these parameters during follow-up were excluded from the study. In the ophthalmic examination, BCVA was measured with a Snellen chart, and intraocular pressure was evaluated with a Goldmann applanation tonometer.

**Fig. 2:**
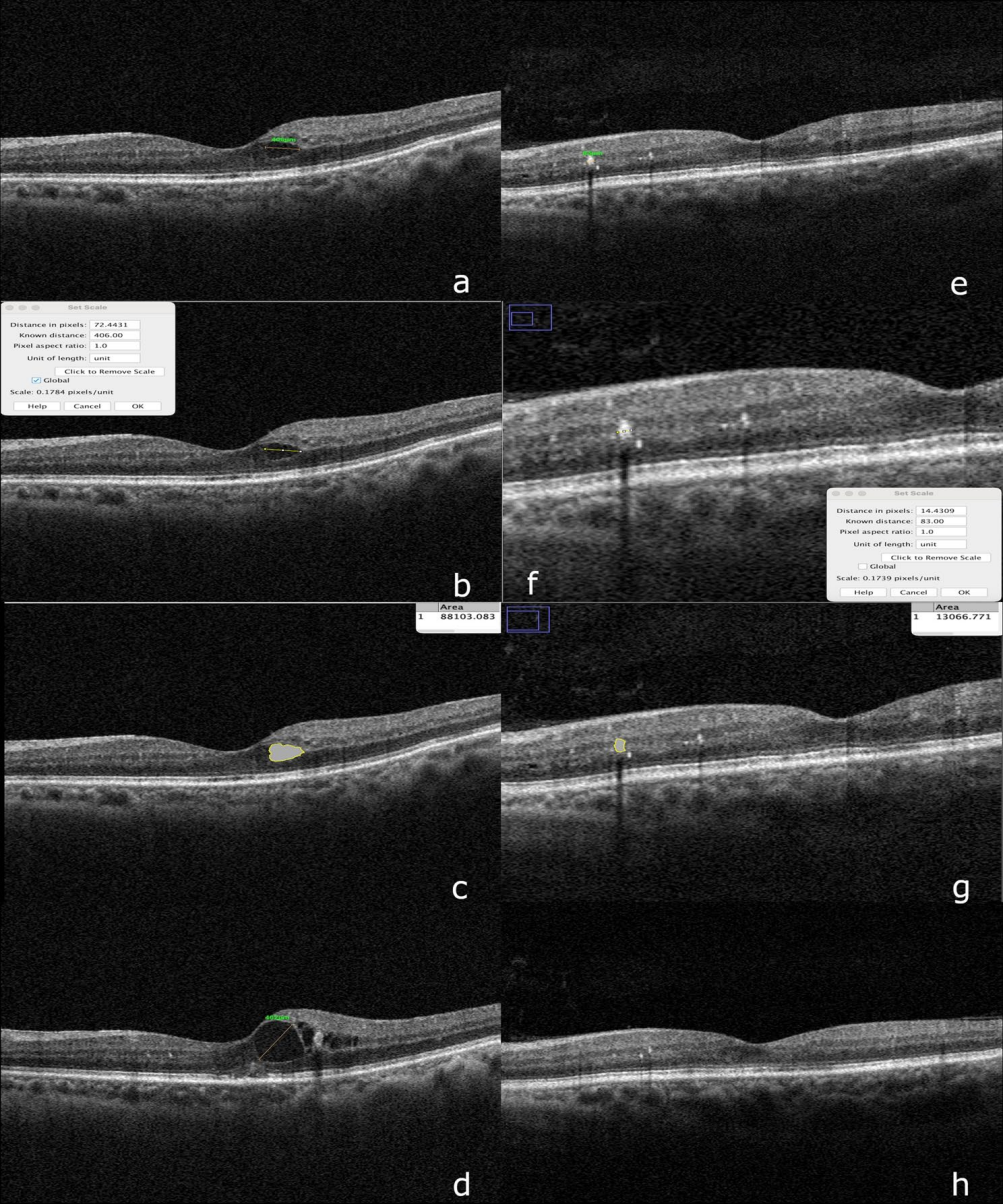
Representative OCT images demonstrating intraretinal cyst and exudate measurement using Fiji ImageJ. Steps using Fiji ImageJ: (**a**) Measurement of intraretinal cyst diameter on a baseline OCT scan. The initial cyst diameter in a patient with NPDR was determined using the OCT device’s distance tool. All B-scans were reviewed, and the widest section was selected for evaluation. (**b**) Calibration of the same cyst in Fiji ImageJ using µm units. The pixel-to-micrometer ratio was derived from the OCT image to ensure accurate subsequent measurements. (**c**) After magnification, cyst borders were manually traced in Fiji ImageJ and the area automatically calculated in µm². Careful contouring improved accuracy. (**d**) Follow-up OCT of the same patient without CaD treatment showed marked cyst enlargement, illustrating progression in the absence of therapy. The patient was advised intravitreal injection and excluded from further follow-up. (**e**) Horizontal diameter measurement of a retinal exudate on the OCT scan of another patient, using the section with the largest appearance. The scale bar was aligned with lesion edges to obtain accurate dimensions. (**f**) The same exudate was calibrated in Fiji ImageJ to µm units. Measurements were aligned to anatomical landmarks for consistency. (**g**) The region was magnified, manually delineated, and the exudate area computed automatically in µm², enabling precise quantification. (**h**) Follow-up OCT of the same patient showed significant regression of exudates. As lesions were largely resolved, maximal diameter could not be measured. At least three anatomical landmarks were used to align imaging planes with baseline scans. Residual small exudates were visible in one section, with posterior hyaloid continuity preserved and posterior shadowing observed due to near-complete resorption

For OCT analyses, the maximum diameter and the area of ​​IRC and IRE were measured using the AngioVue RTVue XR Avanti OCT device (Optovue, CA, USA). The obtained OCT images were analyzed with Fiji ImageJ software, and the measurements were converted to real-world units for detailed evaluation (Fig. 2).

During image analysis, all OCT scans were reviewed by a single experienced retina specialist (B.K.). To minimize potential observer bias, the evaluator was masked to patient group allocation, clinical information, and follow-up time points. All images were anonymized and presented in a randomized order using a standardized coding system before manual delineation of lesion borders in Fiji ImageJ. This masking procedure was consistently applied throughout the quantitative assessment of intraretinal cysts and exudates. During the evaluation, images with a high probability of containing artifacts were excluded from the analysis. In cases where IRE and/or IRC were detected in more than one slice, a standard analysis protocol was applied by selecting the slice with the maximum exudate/cyst diameter. Lesion dimensions were recorded according to the maximum axial diameter using the built-in measurement tool in the OCT software. The pixel-to-micron ratio for images with different pixel resolutions was calibrated using Fiji ImageJ (version: ImageJ2 2.14.0/1.54f). Images were enlarged, IRC/IRE borders were manually marked, and real-world size calibration was performed using the ‘Set Scale’ feature. The same lesion slices were re-evaluated in follow-up images.

The primary endpoint of the study was defined as a 20% decrease in IRC/IRE dimensions measured by OCT in treated eyes. Changes in these parameters were assessed by comparing pre- and post-treatment measurements. Secondary endpoints included the development of CSDME and detection of neovascularization in the optic disc or retinal periphery. In addition, all adverse events occurring during the study, whether related to the study or treatment, were recorded.

### Statistical analysis

Data were analyzed using SPSS 29.0.2.0 (IBM Corp., Armonk, NY, USA) software. Continuous variables are presented as mean ± standard deviation (SD). A normality test was applied to determine whether the parameters in each group were normally distributed; the Shapiro–Wilk test was used since there were 33 participants in each group. Repeated Measures ANOVA was used for normally distributed data, and the Friedman test was used for non-normally distributed data to evaluate changes in three measurement points during the 6-month period for each group. Two-Way Repeated Measures ANOVA was used as a parametric test to evaluate changes over time between groups, and the Mann–Whitney U test was used for non-parametric comparisons. Baseline and control time parameters of four independent groups were compared using one-way ANOVA or the Kruskal–Wallis test. Pairwise comparisons with Bonferroni correction were performed to compare data from two groups at a single time point. *P* < 0.05 was considered statistically significant.

## Results

Demographic data of the 132 patients ultimately evaluated in the study are presented in Table 1. Comparison of demographic and baseline characteristics between the groups revealed no significant differences, except for hemoglobin A1c levels. Among the study groups, the subgroup receiving CaD with IRCs demonstrated the highest HbA1c levels. Post-hoc analysis using the Tukey test confirmed that this difference was statistically significant compared to all other cohorts (*p* < 0.05).Despite the baseline difference, the change in HbA1c levels over the 6-month follow-up period did not differ significantly among the four groups. Furthermore, there were no statistically significant differences among the groups regarding the distribution of systemic treatments, including antidiabetic medication types (insulin vs. oral agents), antihypertensive drug classes (ACE inhibitors/ARBs vs. others), and lipid-lowering therapies (*p* > 0.05 for all). This homogeneity indicates that the baseline metabolic management and concomitant cardiovascular therapies were comparable across the study cohorts.(Table 1).

**Table 1:** Baseline demographic, clinical, and metabolic characteristics of IRC (+) and IRE (+) Patients Stratified by CaD Use

	IRC(+)/CaD(+) *n* = 33	IRC(+)/CaD(-) *n* = 33	IRE(+)/CaD(+) *n* = 33	IRE(+)/IRE(-) *n* = 33	*P* Value
**Age (Years)**	59.33 (± 6.01)	60.76 (± 7.65)	60.48 (± 5.11)	60.6 (± 5.22)	0.769 *
**Sex (M/F)**	18/15	17/16	17/16	16/17	0.97 †
**HbA1c (%)**	6.81 (± 0.45)	6.65 (± 0.48)	6.47 (± 0.41)	6.43 (± 0.47)	0.003 *
**BMI (kg/m²)**	28.76 (± 2.5)	28.91 (± 1.86)	28.69 (± 2.02)	28.25 (± 2.16)	0.605 *
**BCVA (Snellen)**	0.794 (± 0.12)	0.824 (± 0.115)	0.773 (± 0.107)	0.821 (± 0.102)	0.203 ‡
**Insulin / OAD**	17 / 16	15 / 18	13 / 20	14 / 19	0.775 †
**Antihypertensive Therapy, n (%)**					0.845 †
* None*	5 (15.2)	6 (18.2)	4 (12.1)	5 (15.2)	
* ACEi / ARB*	22 (66.7)	20 (60.6)	21 (63.6)	22 (66.7)	
* Others (CCB*,* Beta-blocker*,* etc.)*	6 (18.1)	7 (21.2)	8 (24.2)	6 (18.1)	
Lipid-lowering Therapy, n (%)					0.912 †
* None*	10 (30.3)	12 (36.4)	9 (27.3)	11 (33.3)	
* Statins / Fibrates*	23 (69.7)	21 (63.6)	24 (72.7	22 (66.7)	
**DM Duration (Years)**	10.7 (± 5.4)	10.21 (± 4.99)	8.7 (± 5.54)	8.36 (± 4.58)	0.19 *

BCVA: Best-Corrected Visual Acuity; BMI: Body Mass Index; CaD: Calcium Dobesilate; DM: Diabetes Mellitus; IRC: Intraretinal Cyst; IRE: Intraretinal Exudate; OAD: Oral Antidiabetic Drug

* One-Way ANOVA Test; † Pearson Chi-Square; ‡ Kruskal-Wallis Test

### Retinal Cyst Area (RCA)

**Fig. 3:**
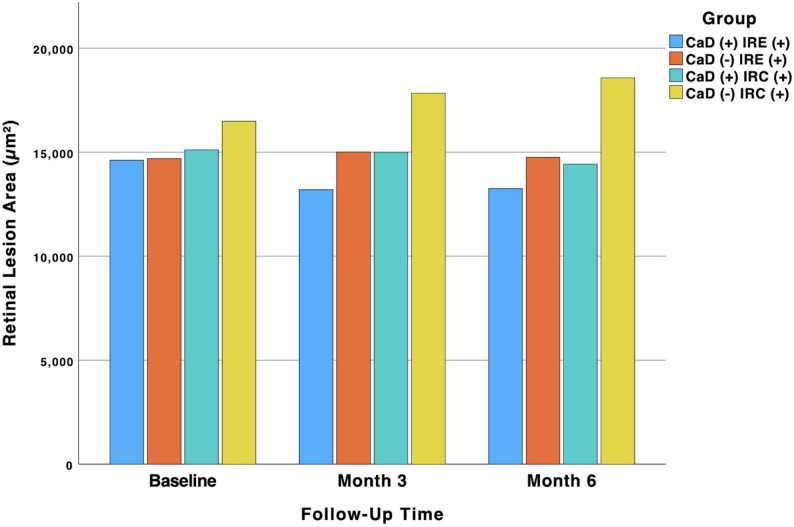
Time-course in RCA and REA across IRC (+) and IRE (+) Groups with and without CaD treatment. CaD: Calcium Dobesilate; IRC: Intraretinal Cyst; IRE: Intraretinal Exudate; RCA: Retinal Cyst Area; REA: Retinal Exudate Area

Figure 3 illustrates the longitudinal changes in RCA over the six-month study period for both the CaD-treated and untreated groups. In the CaD group, a non-significant decrease in RCA was observed at six months (*p* = 0.876). Conversely, the untreated group exhibited a statistically significant increase in RCA over the same period (*p* = 0.002). Intergroup comparisons at specific time points revealed no significant differences at baseline and the 3-month follow-up (*p* = 0.153). However, at the 6-month endpoint, the retinal cyst area was significantly higher in the untreated group compared to the CaD-treated group (*p* = 0.042) (Table 2).

**Table 2:** Longitudinal changes in visual, structural, and metabolic parameters in IRC (+) and IRE (+) Patients with and without CaD treatment

Parameter	Group	Baseline	Month 3	Month 6
BCVA (Snellen)	CaD (+) IRC (+) (*n* = 33)	0.794 (± 0.12)	0.782 (± 0.116)	0.776 (± 0.12)
	CaD (-) IRC (+) (*n* = 33)	0.824 (± 0.115)	0.815 (± 0.13)	0.782 (± 0.136)
	CaD (+) IRE (+) (*n* = 33)	0.773 (± 0.107)	0.767 (± 0.108)	0.755 (± 0.103)
	CaD (-) IRE (+) (*n* = 33)	0.821 (± 0.102)	0.812 (± 0.103)	0.794 (± 0.117)
RCA (µm²)	CaD (+) (*n* = 33)	15,115 (± 3,840)	15,007 (± 7,863)	14,432 (± 7,861)
	CaD (-) (*n* = 33)	16,488 (± 7,127)	17,839 (± 8,062)	18,575 (± 8,792)
REA (µm²)	CaD (+) (*n* = 33)	14,615 (± 4,486)	13,200 (± 3,558)	13,253 (± 7,186)
	CaD (-) (*n* = 33)	14,693 (± 4,754)	15,012 (± 4,753)	15,231 (± 4,970)
HbA1c (%)	CaD (+) IRC (+) (*n* = 33)	6.81 (± 0.45)	6.75 (± 0.44)	6.77 (± 0.38)
	CaD (-) IRC (+) (*n* = 33)	6.65 (± 0.48)	6.63 (± 0.42)	6.64 (± 0.42)
	CaD (+) IRE (+) (*n* = 33)	6.47 (± 0.41)	6.53 (± 0.35)	6.55 (± 0.42)
	CaD (-) IRE (+) (*n* = 33)	6.43 (± 0.47)	6.47 (± 0.41)	6.48 (± 0.42)
BMI (kg/m²)	CaD (+) IRC (+) (*n* = 33)	28.76 (± 2.5)	28.89 (± 2.49)	28.83 (± 2.54)
	CaD (-) IRC (+) (*n* = 33)	28.91 (± 1.86)	28.83 (± 1.77)	28.86 (± 1.83)
	CaD (+) IRE (+) (*n* = 33)	28.69 (± 2.03)	28.63 (± 1.91)	28.7 (± 2.04)
	CaD (-) IRE (+) (*n* = 33)	28.25 (± 2.16)	28.52 (± 2.13)	28.53 (± 2.09)

BCVA: Best-Corrected Visual Acuity; BMI: Body Mass Index; CaD: Calcium Dobesilate; IRC: Intraretinal Cyst; IRE: Intraretinal Exudate; RCA: Retinal Cyst Area; REA: Retinal Exudate Area

Patient eligibility was reassessed at each follow-up visit. The denominators of 109 patients in the CaD group and 203 patients in the control group reflect the initial eligible pools screened prior to propensity score matching and the application of predefined clinical censoring criteria. During follow-up, patients who developed CSDME or centrally involved DME requiring rescue intravitreal therapy were treated as predefined clinical endpoints and were consequently censored from further structural analyses.

At the 3-month follow-up, 7/109 (6.42%) patients in the CaD pool and 22/203 (10.84%) patients in the control pool reached the predefined clinical endpoints of CSDME or centrally involved DME requiring rescue intravitreal therapy (*p* = 0.282). At the subsequent 6-month visit, additional censoring due to these clinical endpoints occurred in 3 of the remaining 102 patients (5.36%) in the CaD pool and 8 of the remaining 181 patients (8.89%) in the control pool (*p* = 0.532). Following these protocols, the final comparative analysis was performed on a balanced cohort of 132 matched eyes (33 per subgroup). (Fig. 1)

### Retinal Exudate Area (REA)

Longitudinal changes in retinal exudate area over the six-month study period are depicted in Fig. 3. The CaD-treated group showed a non-significant decrease in REA at six months (*p* = 0.302). In contrast, the control group exhibited a statistically significant increase in REA during the same period (*p* = 0.002). Pairwise comparisons between the groups at each follow-up time point, as detailed in Table 2, revealed no statistically significant differences in REA at any time.

Additionally, the number of patients excluded from the retinal exudate area analysis due to the development of CSME and fovea-involving DME during follow-up was examined. At the 3-month visit, 6 out of 101 patients (5.94%) in the CaD group and 18 out of 222 patients (8.11%) in the control group were excluded (*p* = 0.648). By the 6-month visit, these exclusion rates were 2 out of 51 patients (3.92%) in the CaD group and 6 out of 86 patients (6.98%) in the control group (*p* = 0.71).

### BCVA

**Fig. 4:**
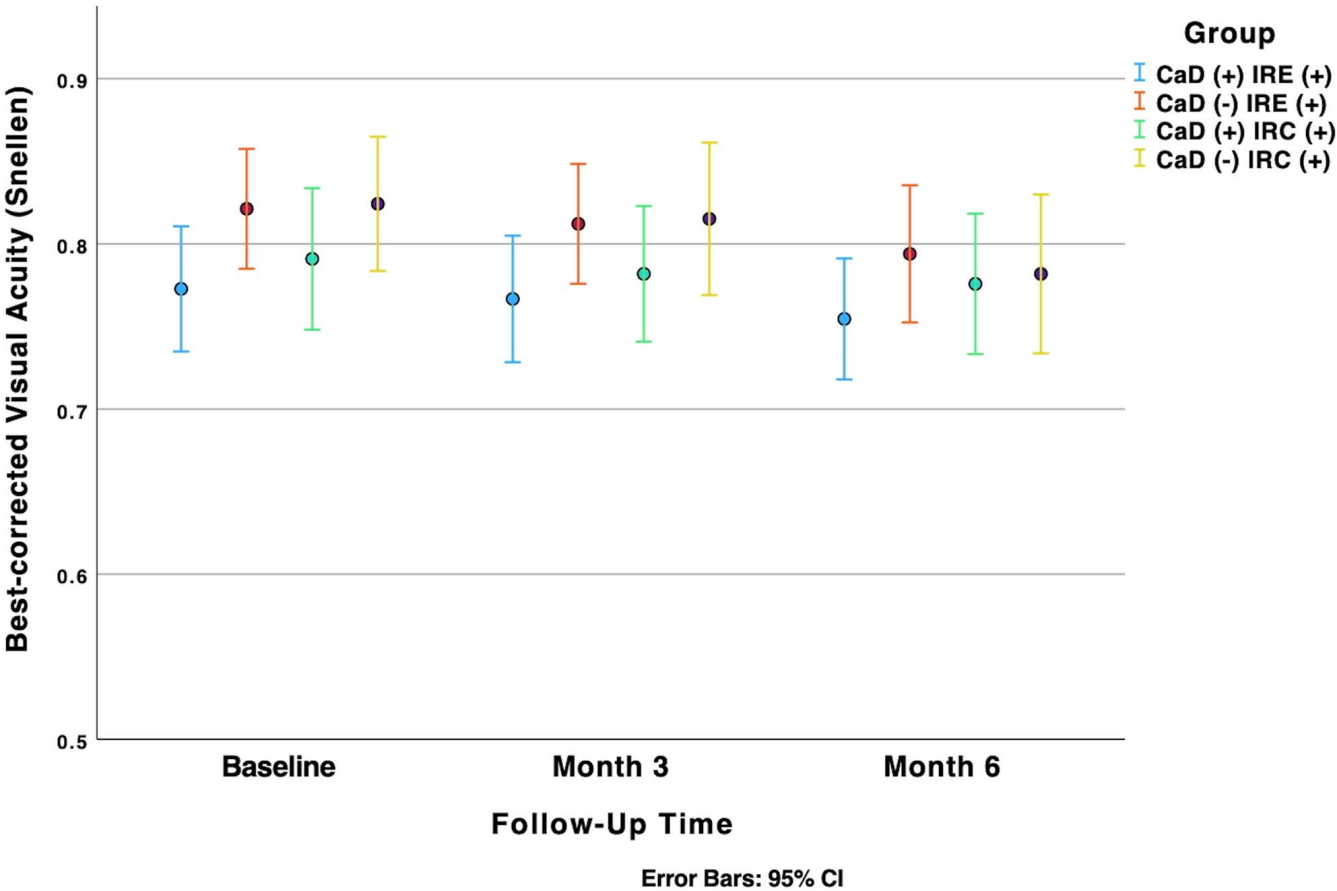
Evolution of visual acuity in IRC (+) and IRE (+) DR patients with and without CaD treatment. CaD: Calcium Dobesilate; DR: Diabetic Retinopathy; IRC: Intraretinal Cyst; IRE: Intraretinal Exudate

Figure 4 illustrates the mean change in BCVA in the treatment group with IRCs from baseline to month 6, which was not statistically significant (*p* = 0.116). In contrast, the control group showed a statistically significant decrease in mean BCVA over the same period (*p* < 0.001). However, pairwise comparisons between the groups at baseline, month 3, and month 6 revealed no statistically significant differences in BCVA (*p* = 0.295, *p* = 0.243, and *p* = 0.833, respectively).

In the subgroup of patients with IREs, the treatment group showed a non-significant decrease in BCVA over the six-month study period (*p* = 0.097), as illustrated in Fig. 3-B. In contrast, the control group in this subgroup exhibited a statistically significant decrease in BCVA (*p* = 0.043). However, inter-group comparisons at baseline, 3 months, and 6 months revealed no statistically significant differences in BCVA (*p* = 0.07, *p* = 0.093, and *p* = 0.167, respectively), as detailed in Table 2.

## Discussion

Advanced DR carries a high risk of serious complications. Those at risk include DME, vitreous hemorrhage, and retinal detachment. These complications can result in significant vision loss even with surgical intervention [18]. Due to this risk of severe vision loss, early diagnosis and treatment of DR are critically important; however, timely intervention in DR can still be more difficult than anticipated and presents significant challenges [19]. In our study, the effects of CaD on IRC and IRE dimensions in NPDR were evaluated, and its potential to alleviate microvascular changes in the early stage was investigated. When examining the pathogenesis of DR, deterioration in vascular structure and function due to increased glucose end products plays a fundamental role. This process progresses with microaneurysms, hemorrhages, exudates, and neovascularization in advanced stages [6]. However, given the complex interplay of systemic and local factors in NPDR progression, the therapeutic impact of CaD remains an area of ongoing debate, necessitating further exploratory analysis. Our findings contribute to this discussion by providing preliminary, hypothesis-generating evidence that supports the potential role of CaD in stabilizing early structural changes, despite the limitations inherent in our study design.

CaD is a widely used agent in various vascular disorders such as chronic venous insufficiency, hemorrhoids, and DR, and is suggested to have antioxidant, anti-inflammatory, and vascular permeability-regulating properties [20–22]. Previous studies have shown that CaD reduces vascular proliferation and permeability and improves retinal microcirculation [23]. In our study, a non-significant decrease in cyst and exudate size was observed in patients treated with CaD, while a significant increase was detected in the control group. These findings suggest that CaD may provide microvascular stabilization and slow lesion progression. Indeed, Zhang et al.‘s meta-analysis reported that CaD reduces microaneurysms, exudates, and hemorrhages, which is consistent with our findings [11]. Wang et al. reported that the combination of CaD and ranibizumab reduces the exudate area and decreases the frequency of injections [24]. However, some studies have failed to show a significant benefit in preventing DME or have reported that the combination with laser photocoagulation is not superior to placebo [20, 25]. These differences are thought to stem from glycemic control, metabolic status, and accompanying comorbidities. In our study, groups were matched according to demographic and metabolic characteristics, and lesion measurements were performed using Fiji ImageJ, aiming to detect microvascular changes more accurately. Notably, although the IRC(+)/CaD(+) group presented with significantly higher baseline HbA1c levels, this cohort demonstrated a stabilization of intraretinal lesions. In clinical practice, suboptimal glycemic control is a primary driver of vascular permeability; thus, the observed structural regression in this metabolically disadvantaged group—contrasting with the progression seen in untreated controls—provides a preliminary clinical signal regarding the potential of CaD to support blood-retinal barrier integrity. However, as the reduction within the CaD group did not reach statistical significance, these results should be viewed as hypothesis-generating rather than definitive proof of efficacy.

The possible effects of CaD are claimed to be through reducing albumin leakage and capillary permeability, oxidative stress, and suppressing aldose reductase activity [26]. Javadzadeh et al. reported that CaD reduced endothelin-1 and CRP levels in DR patients [27]. Similarly, Allain et al. also found a decrease in endothelin levels after CaD treatment [28]. Furthermore, it has been reported that CaD supports endothelial function by increasing nitric oxide levels and inhibits VEGF signaling pathways either alone or in combination with anti-VEGF agents [29, 30]. Njau et al. demonstrated that CaD modulates VEGF signaling by targeting heparan sulfate binding sites [26]. Although the reductions in cyst and exudate size in the CaD group did not reach statistical significance, the marked increase in the control group suggests a potential trend toward microvascular stabilization. While these findings align with mechanistic theories involving the suppression of vascular inflammation and VEGF expression, they should be interpreted as exploratory observations. Furthermore, the lack of significant functional improvement (BCVA) underscores the complex dissociation between structural markers and visual outcomes, which may be influenced by existing neuronal damage or the relatively brief six-month follow-up period.

Our study has several limitations. The retrospective design and relatively small sample size may have limited the power to detect subtle structural or functional changes. A six-month follow-up period is often insufficient to evaluate the long-term course of chronic microvascular diseases. Furthermore, the absence of a placebo group may have prevented the complete isolation of the effect of CaD. In addition, treatment allocation was based on patient preference—often due to the current evidence-based status of the drug—resulting in a non-randomized, self-selected control group and introducing potential selection bias. To minimize its impact, we applied stringent exclusion criteria regarding systemic control (HbA1c, blood pressure, and lipid profiles), thereby reducing the likelihood that the observed progression in the control group was driven by generalized metabolic instability. Nevertheless, selection bias cannot be completely excluded, and the results should therefore be interpreted as exploratory and hypothesis-generating. Our findings are limited to patients with NPDR and type 2 diabetes and cannot be generalized to patients with type 1 diabetes or proliferative DR. Therefore, the results should be interpreted as exploratory and hypothesis-generating. Prospective, long-term, randomized, and masked studies in larger patient populations are needed to confirm these findings.

In conclusion, our findings suggest a potential role for CaD in stabilizing microvascular structures in the management of NPDR. Although the reductions in cyst and exudate sizes did not reach statistical significance, the observed trend toward stabilization contrasts with the significant deterioration seen in the control group. These results, while primarily hypothesis-generating due to the study’s retrospective design and limited sample size, highlight the value of precise quantification using Fiji ImageJ in detecting subtle retinal changes. Our findings suggest that CaD may serve as a cost-effective adjunctive option in early-stage DR; however, large-scale, randomized, and masked prospective trials are essential to validate these preliminary observations and determine their long-term clinical significance.

## Data Availability

The datasets used and/or analyzed during the current study are available from the corresponding author on reasonable request.
